# Understanding the Phase of Responsivity and Noise Sources in Frequency-Domain Multiplexed Readout of Transition Edge Sensor Bolometers

**DOI:** 10.1007/s10909-024-03143-9

**Published:** 2024-05-26

**Authors:** Nicole Farias, Tylor Adkins, Tijmen de Haan, Adrian T. Lee, Anto Lonappan, Megan Russell, Aritoki Suzuki, Praween Siritanasak, Sayuri Takatori, Benjamin Westbrook

**Affiliations:** 1 University of California Berkeley, Berkeley, CA 94720 USA; 2 Institute of Particle and Nuclear Studies (IPNS), High Energy Accelerator Research Organization (KEK), Tsukuba, Ibaraki 305-0801 Japan; 3 International Center for Quantum-field Measurement Systems for Studies of the Universe and Particles (QUP), High Energy Accelerator Research Organization (KEK), Tsukuba, Ibaraki 305-0801 Japan; 4 University of Rome, 00185 Rome, Province of Rome Italy; 5 University of California, San Diego, San Diego, California 92093 USA; 6 Lawrence Berkeley National Laboratory, Berkeley, CA 94720 USA; 7 National Astronomical Research Institute of Thailand, A. Maerim, Chiangmai 50180 Thailand; 8 Research Institute for Interdisciplinary Science (RIIS), Okayama University, Okayama, Okayama 700-8530 Japan

**Keywords:** DfMux, Phase, Noise, Multiplexing, Cosmic microwave background

## Abstract

Cosmic microwave background (CMB) experiments have deployed focal planes with $\mathcal {O}(10^{4})$ transition edge sensor (TES) bolometers cooled to sub-Kelvin temperatures by multiplexing the readout of many TES channels onto a single pair of wires. Digital Frequency-domain Multiplexing (DfMux) is a multiplexing technique used in many CMB polarization experiments, such as the Simons Array, SPT-3 G, and EBEX. The DfMux system studied here uses LC filters with resonant frequencies ranging from 1.5 to 4.5 MHz connected to an array of TESs. Each detector has an amplitude-modulated carrier tone at the resonant frequency of its accompanying LC resonator. The signal is recovered via quadrature demodulation where the in-phase (I) component of the demodulated current is in phase with the complex admittance of the circuit and the quadrature (Q) component is orthogonal to I. Observed excess current noise in the Q component is consistent with fluctuations in the resonant frequency. This noise has been shown to be non-orthogonal to the phase of the detector’s responsivity. We present a detailed analysis of the phase of responsivity of the TES and noise sources in our DfMux readout system. Further, we investigate how modifications to the TES operating resistance and bias frequency can affect the phase of noise relative to the phase of the detector responsivity, using data from Simons Array to evaluate our predictions. We find that both the phase of responsivity and phase of noise are functions of the two tuning parameters, which can be purposefully selected to maximize signal-to-noise (SNR) ratio.

## Introduction

Simons Array (SA) is an experiment located in the Atacama Desert in Chile at an altitude of 5200 m. The goal of Simons Array is to measure the polarization of the Cosmic Microwave Background using $\mathcal {O}(10^{4})$ transition-edge sensor (TES) bolometers in its two receivers, PB-2a and PB-2b [1, 2].

**Fig. 1:**
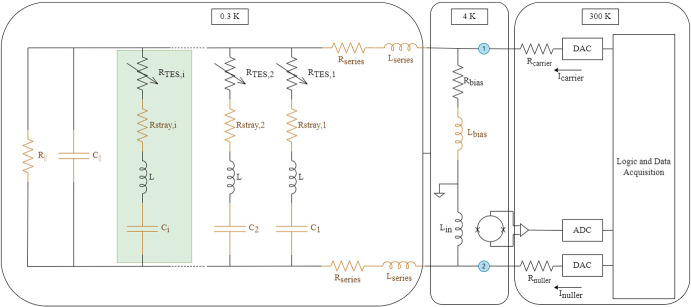
DfMux circuit diagram. Components in beige are fit for in the admittance model. One “leg” of the readout circuit is highlighted in green. $R_{\parallel }$, $C_{\parallel }$, $R_{\textrm{stray,i}}$, $R_{\textrm{series}}$, $L_{\textrm{series}}$ and $L_{\textrm{bias}}$ are parasitic components included in the model, whereas other components are part of the DfMux design. $R_{\textrm{nuller}}$ and $R_\mathrm {{carrier}}$ are stiffening resistances for the nuller and carrier currents

To read out its thousands of detectors, Simons Array utilizes digital frequency-domain multiplexing (DfMux) with a multiplexing factor of $40\times$. In this scheme, each TES detector is assigned a frequency channel by being connected in series with an inductor and capacitor (LC) resonator. The TES resistance, inductor and capacitor form an LCR “leg” with resonant frequency $f_r$, and each TES is voltage biased by a carrier containing independent tones at the different $f_r$. As the optical power on the TES changes, its resistance changes and results in an amplitude modulation of the current through each leg. The signal from the TESs is amplified first by a Superconducting Quantum Interference Device (SQUID) Array Amplifier (SAA), and then by room temperature electronics before being demodulated. The single-stage SAAs are of the NIST SA13a design, which was selected for Simons Array after its characterization reported in [3]. The 210 SAAs are located in the 4 K stage to limit the thermal load onto the sub-Kelvin stages. To linearize the SAA output and maximize dynamic range, current through the SAA is nulled using the technique of Digital Active Nulling (DAN)[4]. The nulling of currents at the SAA junction also has the benefit of suppressing the SAA input inductance in DAN’s effective circuit admittance. A simplified circuit of the readout scheme is shown in Fig. 1, where some parasitic components are included.

Two-level states in the LC resonator chips of Simons Array were found to induce phase noise [5]. While the phase noise was initially expected to not have a significant impact on the SNR of SA detectors, its actual contribution hadn’t yet been quantified. In this article, we report on a characterization of the phase associated with SA’s measured signal and noise sources, including that of two-level system (TLS) noise from the resonator chips. By understanding the phase behavior of each noise source, it is possible to predict and minimize their impact on the experiment’s sensitivity. This characterization is not only important for SA, but for other experiments that use DfMux technology, such as the LiteBIRD satellite mission[6].

## Methods

To quantify how the phase of each noise source affects the SNR, three steps were taken. First, we reviewed how the *I* and *Q* phase are defined in DfMux. Then, we modeled and measured the phase of the TES’s responsivity to an optical signal. Finally, we investigated the phase associated with each of the major noise contributors in the instrument. All data presented in this article were taken while the telescope was stationary and observing the “stimulator” signal. The PB-2a stimulator is a thermal source at a temperature of roughly 700 ^∘^C that is chopped at a frequency of 5 Hz. It is typically used in SA a part of the calibration routine both before and after science scans.

### How Phase is Defined in DfMux

When the current signal gets demodulated by room temperature electronics, it is decomposed using I/Q demodulation. The *I* (in-phase) component is aligned to the phase of the carrier $\angle I_\textrm{carrier}$ and by consequence to the phase of admittance $\angle Y$, where $Y=I_{\textrm{carrier}}/V_b$ and $V_{\textrm{b}}$ is the voltage bias. The phase of the demodulation is chosen to be anti-aligned with the phase of the nuller signal to ensure cancellation at the SQUID summing junction. Every time that the carrier current is adjusted to either bring the TES into, or out of, their superconducting transitions, the phase of the carrier is adjusted to ensure that $<Q>=0$.

### Phase of Responsivity

Fluctuations in optical power induce changes in the TES’ resistance which in turn result in changes in the current measured by DfMux readout. Thus, the phase of a response to an optical signal is aligned with $\angle (di(t)/dR(t))$, which we’ll now refer to as the phase of responsivity. To understand some of the parameters that can impact the phase of responsivity, we can start with a simplified model where each parallel leg, consisting of an inductor, a capacitor and a TES, (the green shaded region in Fig. 1) can be simplified as an RLC circuit driven by a voltage bias with frequency $\omega$. If we neglect stray resistances, the total complex current through the circuit (aligned with *I*) is then:

1 $$\begin{aligned} i(t) = V(t) \frac{R(t)}{|Z|^2} + j V(t) \frac{\omega L - 1/(\omega C)}{|Z|^2} \end{aligned}$$

If the AC circuit is on-resonance, $\omega L = 1/(\omega C)$ and the circuit reactance is zero. In this case, changes in resistance are completely aligned with the I component of the demodulated signal. If the circuit is off-resonance, the responsivity is out of phase with component I by:

2 $$\begin{aligned} \phi _{\textrm{res}} = \arctan {\left( \frac{\omega _b L - 1/(\omega C) }{R} \right) } \end{aligned}$$

In reality, even when detectors are biased at the resonators’ resonance frequencies, the impedance from neighboring TES legs and from parasitics affect the discrepancy between responsivity phase and admittance (*I*) phase. Each circuit of resonators and TESs can be fit to a model containing these expected impedances, and this model can be used to predict the responsivity phase of its associated TESs. A diagram of the modeled circuit is shown in Fig. 1, where the measured impedance is the DAN effective impedance between points 1 and 2 in the circuit. The beige components are the parameters that are fit for in the model, while the other components are fixed. Note, however, that the nulling of the current through $L_{in}$ via DAN creates a virtual ground across the SAA input inductance, suppressing its impact on modeled phases. The analytical expression for the admittance model is given in eq. 3. $R_{\textrm{bias}}$ is fixed to be $30\ m\Omega$, while $L_{\textrm{bias}}$ is fit for and is expected to arise from the tracelines in the PCBs. $C_{\parallel }$ and $R_{\parallel }$ are lumped elements that represent the equivalent capacitance and resistance of other (unintended) current paths in parallel with the TES-resonator pathways. All of the resonators in the circuit have the same inductance *L* fixed to be $59.6\ \mu H$. The capacitance of the resonators, $C_{i}$, varies and is fit for in this model. $Z_{\textrm{s}}$ is meant to capture parasitic impedances in series with all of the resonator and TES pathways. Typically, these measurements are done with the TES in a fully superconducting state, so $R_{\textrm{TES,i}} = 0$. $R_{\textrm{stray,i}}$ is the fit stray resistance in a given TES-resonator pathway. *N* is a normalization parameter.

3 $$\begin{aligned} \begin{aligned}&Y = N/|1+\left( Z_{\textrm{s}}+\frac{1}{j\omega C_{\parallel }+\frac{1}{R_{\parallel }}+\sum _{i=1,2,...,N} \frac{1}{j\omega L+1/\left( j\omega C_{\textrm{i}}\right) +R_{\textrm{TES,i}}+R_{\textrm{stray,i}}}}\right) \frac{1}{LR_{\textrm{bias}}}| \\&Z_{\textrm{s}} = R_{\textrm{series}} + j\omega L_{\textrm{series}} \\&LR_{\textrm{bias}} = j\omega L_{\textrm{bias}} + R_{\textrm{bias}} \end{aligned} \end{aligned}$$

Following [7], we measured the DAN effective impedance by separately sweeping both carrier and nuller signals across our readout bandwidth and calculating the relative admittance between the two. The measured impedance between 1.5 and 4.5 MHz was used to fit the parameters in the model. We used this circuit model to predict phase shifts in PB-2a’s readout which were then compared to the measured phases between *I* and the TESs’ responsivity in data from PB-2a. This was done by changing the *I* and *Q* basis via Eq. 4 to maximize the measured peak in the $I'$ amplitude spectral density (ASD) from the stimulator signal at $5 \pm 0.3$ Hz.

4 $$\begin{aligned} I' + jQ' = e^{j\phi } (I + jQ) \end{aligned}$$

### Phases of Noise

The major sources of noise in PB-2a (photon, phonon, readout and Johnson noise) can be decomposed according to their associated phases. Photon noise and phonon noise are power sources on the TES, which are converted to current noises via the detector responsivity. Thus, by definition, these noise sources only contribute to the total noise in-phase with responsivity. Broadband readout noise has no preferential phase and thus generates equal contributions in and out of phase with the TES responsivity. This is the case for SQUID noise and noise from room temperature electronics. There are three noise sources that are expected to be mostly *out of phase* with responsivity.

First, TES Johnson noise is suppressed by the detector’s electrothermal feedback. However, by definition, this suppression does not occur out-of-phase with responsivity [8]. Second, as described in [7], leakage current and power crosstalk from neighboring TESs are expected to be many degrees out-of-phase with the signal phase. Finally, two-level system noise has been seen in lithographed superconducting resonator chips, and is caused by the coupling of the resonator to a thin amorphous solid dielectric layer where two-level tunneling states are thought to exist [9]. This noise is equivalent to a jitter in the resonance frequency, which has manifested itself as an excess noise in the *Q* component of the current in SA hardware utilizing DfMux [5].

It is important for experiments using DfMux that the excess phase noise does not increase noise in the responsivity phase of the detectors. We’ve added to the characterization of noise from [5] by investigating the impact of detector operating resistance $R_{\textrm{frac}}$ and bias frequency onto the phase that minimizes noise. As part of this study, we’ve measured instrument noise when the TES is biased $\pm 450$ Hz off from the LC resonance peak. This induces additional reactances to the readout circuit which cause shifts to the phase of responsivity. This effect can be utilized in an attempt to best align the phase of detector responsivity with the phase that minimizes the noise in the demodulated signal. In addition, the added impedance can increase TES responsivity [10], increasing the overall SNR.

Similarly to how the phase of responsivity is measured in SA TESs, we measure the phase of minimum noise by recursive offline phase rotations until the median of the $I'$ ASD between 6 and 9 Hz has been minimized. This frequency range is chosen to avoid resonances from the stimulator signal at 5 Hz while still capturing noise within the detector’s time response.^1^

## Results

*In this section, all angles are taken with respect to the* *I* *component of the demodulated current.*

### Phase of Responsivity

**Fig. 2:**
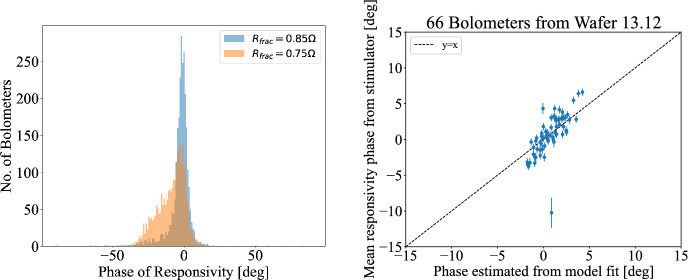
(*Left*) Measured phase of responsivity at different target TES operating resistance. (*Right*) Phase of responsivity predicted by impedance fit (x-axis) and measured from stimulator signal (y-axis) at a target $R_{\textrm{frac}}$ of 0.75 $\Omega$

The phase of the TES responsivity was found to be offset by a few degrees from *I* when the TES resistance is close to its normal value of 1 $\Omega$. As detectors go lower into their superconducting transition, their responsivity phases start to spread further, as shown in the left panel of Fig. 2. This is expected as the reactances in the circuit induce higher phase shifts when the TES resistance is smaller, as suggested by Eq. 2.

An example of the model predicted phase shifts relative to the demodulation phase compared to the measured ones can be seen on the right panel of Fig. 2. The data is from TESs from three separate LC circuits currently in the PB-2a receiver. These three circuits were chosen due to their associated lower Chi-squared in the fit of the measured impedance to the model of Fig. 1, which resulted in better agreement with the responsivity phase predictions. In other words, accurate modelling of the readout impedances allows us to predict the phase response of our detectors, which is not necessarily aligned with *I*.

### Offset Between Phase of Responsivity and Phase of Noise

**Fig. 3:**
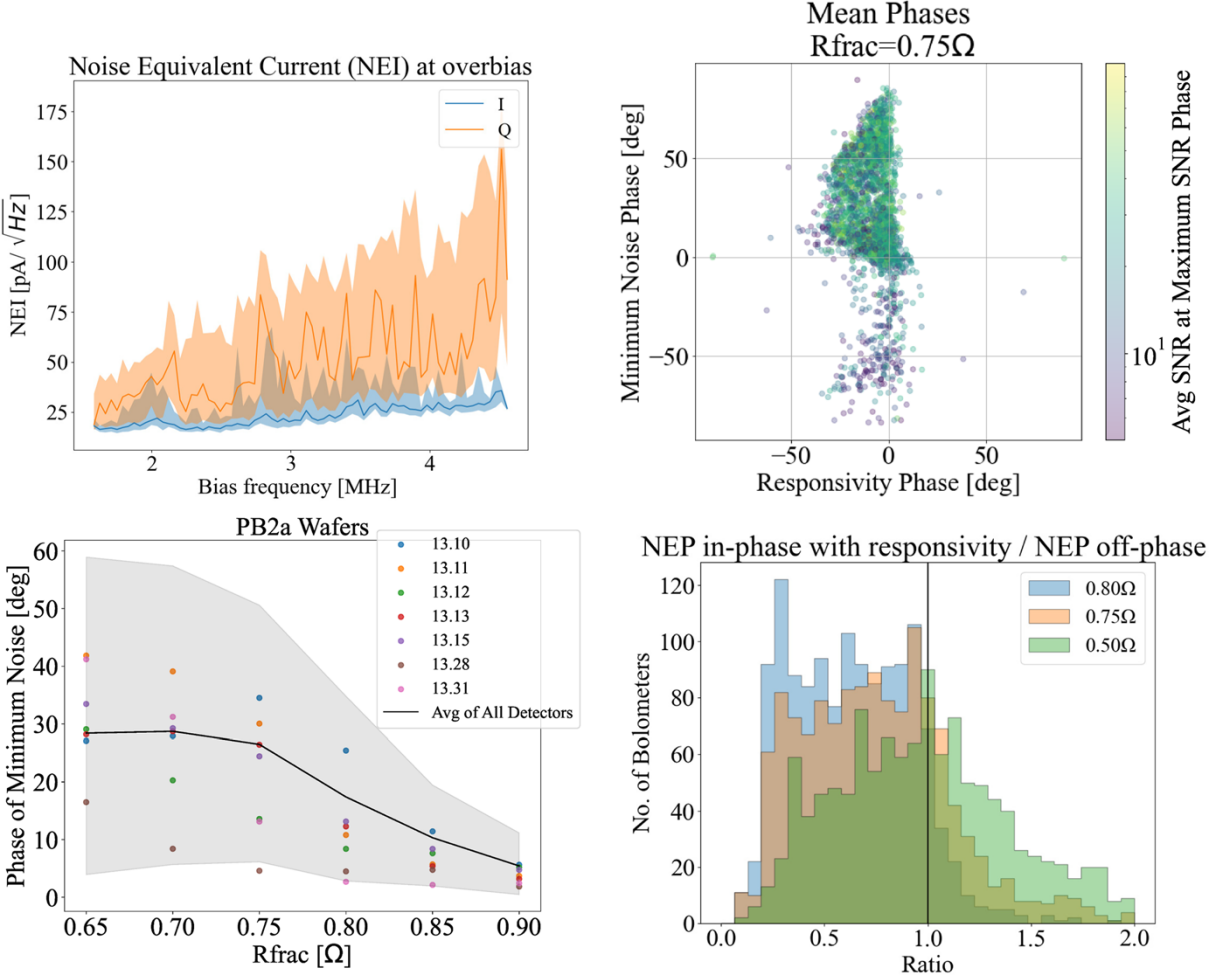
(*Top Left*): Noise-Equivalent Current (NEI) of saturated detectors in *I* and *Q* components of the demodulated current. (*Top Right*): Phases that maximize signal and minimize noise for detectors tuned to an $R_{\textrm{frac}}$ of 0.75 $\Omega$, averaged over multiple observations. Colormap indicates the SNR of each detector. (*Bottom Left*): Phase that minimizes detector noise as a function of $R_{\textrm{frac}}$. Each color represents a different detector wafer and the average over all detectors is represented by the black line. (*Bottom Right*): Ratio of Noise-Equivalent Power (NEP) in phase with detector responsivity to NEP out of phase at different target $R_{\textrm{frac}}$ values

When detectors are saturated and not in transition, we see a larger noise amplitude in the *Q* component of the demodulated current than in the *I* component, as shown in the top left panel of Fig. 3. In this state, the phase of minimum noise would be aligned with *I*. However, when detectors are in transition, more factors can affect the phase of dominant noise. As discussed in the previous section, the responsivity is not necessarily aligned with *I*. In addition, when detectors are in transition, noise sources aligned with responsivity contribute to the noise budget. Furthermore, the decreased TES resistance can change the impact of the phase shifts induced by circuit parasitic impedances on the noise.

The top right panel of Fig. 3 shows a comparison between the phases that maximize TES responsivity versus the phase that minimizes detector noise when detectors are in transition at an $R_{\textrm{frac}}$ of 0.75. The colormap shows that even for detectors with average or high SNRs, there is a large spread in the phase that minimizes noise which does not align with the phase that maximizes responsivity. We don’t believe this spread is due to measurement uncertainty as individual TESs show small variance across many observations. This phase of minimum noise was found to be a function of $R_{\textrm{frac}}$ and of detector wafer, as shown in the bottom left panel of Fig. 3. The black line in this plot shows the average over all detectors and shows a general trend that the phase of minimum noise shifts further from the demodulation phase at lower $R_{\textrm{frac}}$. The shaded region represents the region between the 20th percentile and 80th percentile of the distribution of all detectors’ minimum noise phases. There are fewer observations for any individual detector at lower $R_{\textrm{frac}}$ which partially accounts for the widening of the total distribution. The carrier phase was re-aligend every time detectors are tuned to a specified $R_{\textrm{frac}}$.

Finally, the bottom right panel of Fig. 3 shows the ratio between the noise equivalent power (NEP) of each detector in phase with responsivity to NEP out of phase with responsivity. When $R_{\textrm{frac}}$ is high, the dominant noise in most detectors is out of phase with responsivity. However, as detectors go lower into the transition, the ratio starts to increase and for a larger number of detectors, the dominant noise sources are in phase with responsivity.

### Off-Resonance Biasing

**Fig. 4:**
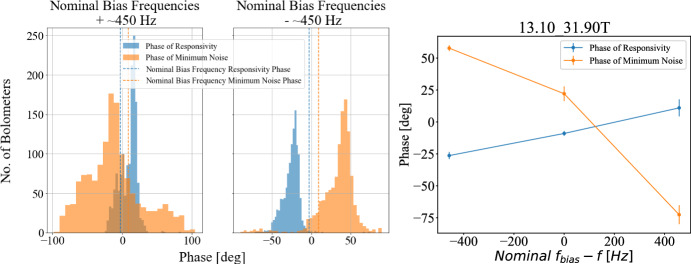
(*Left*): Observed responsivity and noise phases when detectors’s bias frequencies are shifted by ±450 Hz from resonance peak. (*Right*): Changing bias frequency can enable aligning the phase of maximum signal and of minimum noise. Data points come from a single detector and were averaged over at least two distinct observations

Biasing away from the resonance that maximizes the admittance described in 2.2 will further shift the phase of responsivity from the phase of circuit impedance. The left panel of Fig. 4 shows the responsivity phase shift for many different SA TESs, whereas the right panel shows an example of a single TES’s phases of maximum signal and of minimum noise at different bias frequencies. The point of intersection between the two lines shows where the offset between those two phases can be minimized by optimizing the bias frequency, indicating that biasing detectors slightly off-resonance is a strategy that can be used to align maximum signal and minimum noise phases in SA TESs.

## Conclusion

We investigated the responsivity and noise phase behavior in the DfMux scheme implemented in the Simons Array’s PB-2a receiver. Both phases were found to depend on TES tuning parameters such as $R_{\textrm{frac}}$ and bias frequencies, and we were able to predict the responsivity phase behavior by characterizing the impedances in the cold readout. Adding a small offset to the detectors’ bias frequency resulted in shifting both responsivity and noise phases. This effect could be used to align phase of maximum responsivity and phase of minimum noise.

## Footnotes

1 The effective time constants for the two observing bands of PB2a, 90 and 150 GHz, are typically 4 and 10 ms, respectively.
